# Smart Portable Devices Suitable for Cultural Heritage: A Review

**DOI:** 10.3390/s18082434

**Published:** 2018-07-26

**Authors:** Federica Valentini, Andrea Calcaterra, Simonetta Antonaroli, Maurizio Talamo

**Affiliations:** 1 Sciences and Chemical Technologies Department, Tor Vergata University, via della Ricerca Scientifica 1, 00133 Roma, Italy; simonetta.antonaroli@uniroma2.it; 2 INUIT Foundation Tor Vergata University, via dell’Archiginnasio snc, 00133 Roma, Italy; andrea.calcaterra@uniroma1.it (A.C.); Maurizio.talamo@uniroma2.it (M.T.)

**Keywords:** Sensors, Cultural Heritage (CH), integrated sensor arrays, actuators, movable devices, ICT, mobile Laboratory, IoT, air quality control, analytical diagnosis, in situ restoration, nanomaterials, graphene, graphene oxide (GO)

## Abstract

This article reviews recent portable sensor technologies to apply in the Cultural Heritage (CH) fields. The review has been prepared in the form of a retrospective description of the sensor’s history and technological evolution, having: new nanomaterials for transducers, miniaturized, portable and integrated sensors, the wireless transmission of the analytical signals, ICT_Information Communication Technology and IoT_Internet of Things to apply to the cultural heritage field. In addition, a new trend of movable tattoo sensors devices is discussed, referred to in situ analysis, which is especially important when scientists are in the presence of un-movable and un-tangible Cultural Heritage and Art Work objects. The new proposed portable contact sensors (directly applied to art work objects and surfaces) are non-invasive and non-destructive to the different materials and surfaces of which cultural heritage is composed.

## 1. Introduction

At the state of the art, humidity, temperature sensors [1,2], gas and particulate matter sampler devices [3,4] and smart apparatuses, specific for the monitoring of the biofilms [5,6] represent the most applied tools for the air quality control of indoor [7] and outdoor [8] environment, where Cultural Heritage are located. Recently, all these devices have been designed as new miniaturized tools in order to be ready for in situ diagnosis of Cultural Heritage, especially to be carried out on unmovable and intangible art work surfaces. The sensor market is very appealing especially because several applications used smart movable sensors, such as: medical health care applications [9], sport performances, with dedicated smartphone app [10], food quality and food packaging fields [11] and also the climate global changes [12] forecasts. Considering the global market forecasts, sensors to apply on the damaged Cultural Heritage surfaces have not been reported yet [13]. This aspect certainly represents a limitation or drawback when unmovable and intangible CH need to be investigated. Until now, conventional portable sensors have been dedicated to the monitoring of the air quality of the indoor environment, where CH are located. At the state of the art, there are no sensors properly designed for in contact mode monitoring of the damaged surfaces of the art work objects. For this purpose, scientific research needs to develop innovative miniaturized tools able to work directly in contact with CH surfaces, not only when unmovable art work objects are considered but also to deeply understand the complex damages phenomenon. In fact, the latter results in a complex combination of environmental phenomena, which mainly affect the surface of Cultural Heritage but also inner processes, due to the raw original materials of which the CH objects are composed. For this purpose, a very promising challenge in the CH field could be the assembly of tattoo sensors [14,15] for surface analysis and also nanomotors and nanomachines, able to work in the bulk of CH objects. In the first case, the new tattoo sensor prototypes could be able to monitor the chemical-physical modifications that occur on the surface of art work objects, because of their molecular interaction with the polluted environment. In the second case, nanomotors could be suitable to identify and quantify the bulk damages, strictly related to the natural aging of the original materials of CH. Additionally, when nanomachines detect the deterioration events into the bulk areas of art work objects, they also behave like smart actuators, able to release, in a controlled manner, the restoration and conservation reagents. In this way, not only in situ diagnosis could be performed but also *in loco* restoration/conservation treatments can occur, directly in contact with the CH objects. This could represent the future scientific challenge in the CH field! To achieve this goal, new nanomaterials with improved performances are required, such as: graphene like derivatives, [16], metallic nanoparticles [17], nanostructured polymers [18] and nanocomposites [19]. All of them exhibit excellent chemical-physical properties [20], if compared with the conventional sensor materials, such as: higher electrical conductivity [21], higher thermal stability [22], lower Young’s modulus (that is essential to obtain flexible and stretchable electrodes/sensors [23,24]) and fascinating/superior optical features (useful for the fabrication of transparent electrode/sensor support [25,26]). In addition, all the cited nanomaterials, also result highly eco-friendly materials (having a low environmental impact) and biocompatible systems toward “end-users” (mainly restorers and conservator scientists, in the CH field), especially after their selective engineering, which makes them suitable for the final applications. All these considerations are not only future predictions because nanomaterials already exist, printing innovation technologies [27], which are now a well-established reality. Especially, the printing nanotechnologies are suitable to fabricate miniaturized sensors with highly compatible materials [28,29] with those of which the CH objects, are made [30]. For examples, paper support and several other natural textiles have already been explored to make a new generation of electrochemical sensors [31,32,33,34,35,36,37]. The resulting electrochemical devices exhibit enhanced electrical conductivity [38], mechanical stability [39] and compatibility [40] especially towards the art-work surfaces, respecting the fundamental criteria of not invasiveness [41] of the CH substrata. 

According to the state of the art, describe in the introduction section, the authors decide to organize the review into two sections, reported below:

- The first one, dedicated to a complete overview of the portable humidity sensors [42], temperature probes [43], pH devices [44] and gaseous/particulate matter sampler/collectors [45,46], which are necessary to perform the air quality control of the indoor/outdoor environment, where CH are located. Additionally, a paragraph is also dedicated to the new generation of the portable devices, suitable to work, directly in contact with the art work surfaces. A summary of the main challenges, research needs, limitation and drawbacks of the sensors designed for CH, are shown on Table 1.
- The second section considers an engineered integration of movable sensors and portable actuators (i.e., nanomachineries) could represent the first proof-of-concept of a mobile Laboratory/Lab, suitable to carry out in situ diagnosis and *in loco* remediation/restoration of art work surfaces. It is necessary for the movable Lab to be interfaced with the most innovative ICT and IoT technological systems, to be completely autonomous in experiments and to process big data.

## 2. Section I: The Portable Humidity, Temperature, pH Devices and Pollutants Samplers

Recently, in the sensors field application the miniaturization trend seems to be very promising because in situ diagnosis and *in loco* remediation would be desirable. In the first section, several different miniaturized sensors are presented, some of the most important applications in museums, archives, libraries and galleries are also reported (as cases of study) and finally, a critical analysis of their performances, has been also described.

**Figure 1:**
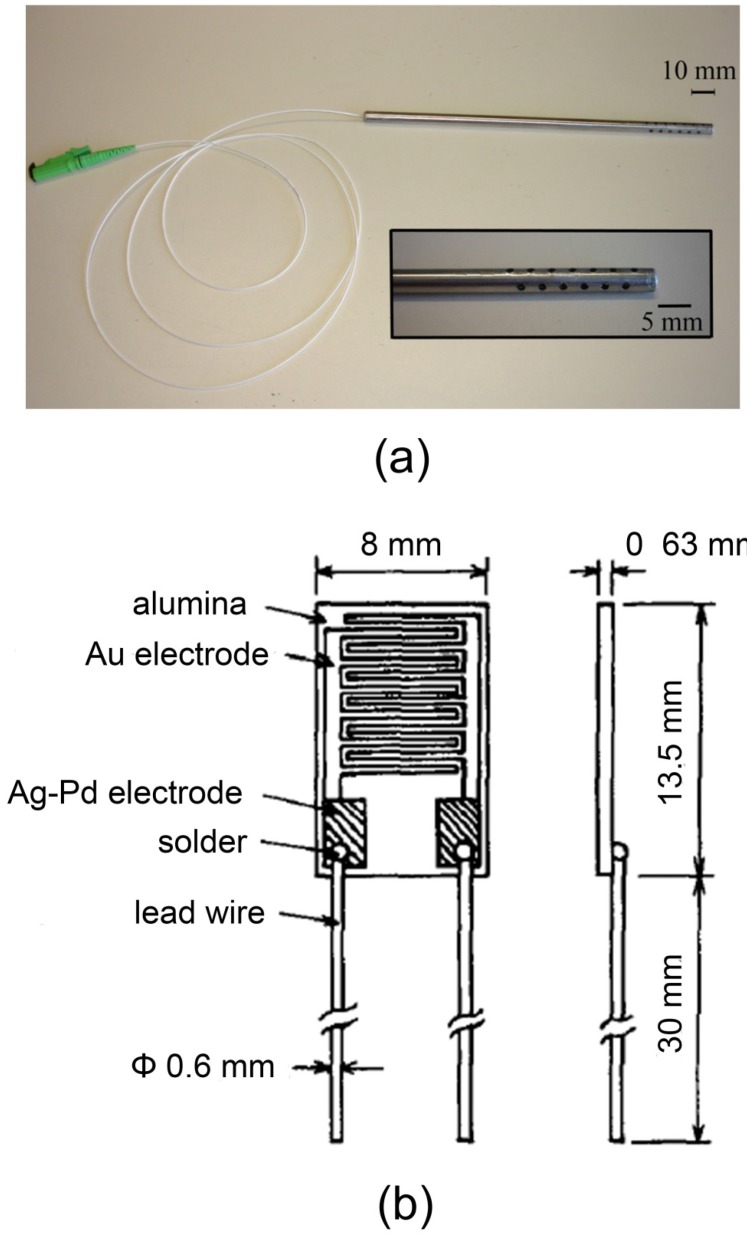
(**a**): Prototype of the improved RH optical sensor equipped with a protective stainless steel tube [48]; (**b**): schematic view of the Hument HPR type humidity sensor device [57].

Miniaturized optical humidity sensors. Recently the fiber based devices seem to be very powerful tools for the fabrication of portable humidity sensor and also to provide the multiplexing of temperature, humidity, displacement, pressure, pH value, high magnetic field and acceleration probes) into the same optical fiber, minimizing the multiple conventional cabling [47]. Especially, Relative Humidity %, temperature and pH levels represent the main parameters to monitor in museums, to establish the conservation status of CH objects. Here, the authors report an organic-silica hybrid material as a water sensitive layer of optical fiber transducers. Especially, the di-ureasil humidity sensitive layer was chemically deposited on the transducers and it consisted of polyether chains with 600 g·mol^−1^ (labeled as d-U(600)), as the average molecular weight covalently linked to a siliceous inorganic skeleton by urea bridges [48]. The average diameter of the deposited d-U(600) sensitive layers, on the optical transducers, ranging from 375 ± 5 μm to 591 ± 5 μm (Figure 1a). Its volume reversibly depended on distinct levels of RH, that in this way, was quantified but with low sensitivity. This latter analytical parameter was significantly improved by using reduced graphene oxide [49] to coat the fiber transducer. In this case, the detection of the output signal was carried out considering the power leakage, at resonance wavelength. In fact, when the hollow core fiber was coated by using reduced graphene oxide, a Fabry–Perot resonator was obtained in the cladding region [49]. The leaky mode of the guided light was reached at the resonant wavelength of the Fabry–Perot resonator, which results in a lossy dip in the transmission spectrum of the hollow core fiber. Considering the tunable refractive index of the reduced graphene nano sheets, the humidity was quantified using the transmission intensity of the lossy dip. Experimental data showed an improved sensitivity up to 0.22 dB/% RH. In addition, the reduced graphene based humidity optical sensor also exhibited good repeatability, fast response time and low temperature cross-sensitivity, resulting eligible for in situ monitoring of the conservation status of CHs.

According to this, a practical testbed, applied in a museum called Fortaleza São Tiago, located in Madeira Island (Portugal), is reported here to show the sensor performances. Three Crossbow [50] mica2 motes, equipped with a MTS400CA data acquisition board and a mote equipped with a mib520 board (representing the base station) were installed in this museum. The MTS400CA device measured all these parameters: the ambient light, the relative humidity, the temperature, the 2-axis-accelerometer and the barometric pressure. This board contained a conventional fiber based humidity sensor and the Sensirion SHT11, as temperature probe. This was an example of multiplexing sensor, suitable to measure temperature, whose values ranging from −10 to +60 °C and the corresponding humidity levels, ranging from 0 to 90% RH. The acquired data showed the typical variations of temperature and humidity parameters, throughout the day and night, in a single room of the museum. In particular, experimental results showed that the temperature ranged about 2 °C, whereas the relative humidity ranged about 6% RH. According to the museum’s managers, these variations resulted as typical values, especially during winter time. Instead, the parameters exhibited much higher values in the summer, accordingly to the day and night periods, or when the lights were turned on/or off. Experimental results demonstrated that the portable Sensirion SHT15 device (which is a multiplexing sensing platform combining the Sensirion SHT11, as temperature probe, with a conventional fiber humidity sensor) was suitable for monitoring the thermoigrometric conditions that agreed with the standard values, established according to the *UNI 10829 (1999)* and *UNI 10969 (2002)* references regulation for indoor environments.

Miniaturized capacitive humidity sensors. MEMS (Micro-electro-mechanical systems) technology and nanomachining techniques provide a wide impact on the miniaturization of sensors and portable integrated devices. Lee et al. [51] developed a novel fabrication process for Pt Resistor Temperature Detectors (RTD, OMEGA Engineering Inc., Taoyuan, Taiwan), combined with a micro-cantilever, covered with a water sensitive polymeric layer (labelled as organic MEMS). In this case, a capacitive detection mode, occurred applying the MEMS devices. In the Cloisters, the medieval branch of the New York Metropolitan Museum of Art [52], wireless MEMS based sensors were successfully installed. The temperature transducer was a semiconductor diode element (TMP112 with 12-bit resolution, Texas Instrument, Dallas, TX, USA) with an accuracy of 0.5 °C. The air temperature probe was used to study the thermal stratification along the height of a gallery and also the slight temperature gradients between different galleries. The experimental results showed that the MEMS devices continuously monitored the temperature profiles in the museum, for the whole year. The measured value of 20.1 °C agreed with the reference values described by *UNI 10829 (1999)* and *UNI 10969 (2002).* Temperature was strictly related to the relative humidity content and changes in indoor relative humidity could be detected only when the difference between the outdoor and indoor humidity, was relatively large. The relative humidity (RH) sensor was a capacitive tool (SHT21, 14-bit resolution, Sensirion, Stafa, Switzerland) with an accuracy of ±2%. An influx of outside air, due to the opening and closing of the museum doors, involved an entry of moisture, which may lead to undesired condensation, on cooler art work objects. Experimental results demonstrated the great advantage of monitoring thermoigrometric conditions across the whole building. The thermoigrometric parameters quantification, was suitable to understand the air stratification and the environmental connections, between rooms and the insulating properties of the building walls, windows and doors. To improve the sensitivity of the capacitive humidity sensors, miniaturized electrodes, modified with G-O nano sheets [53], were assembled. The resulting devices showed a sensitivity 10 times higher than that exhibited by the humidity sensors, fabricated with water sensitive polymeric layers [54]. The G-O sensors exhibited a fast response time (less than 1/4) and recovery time (less than 1/2) if compared with the organic MEMS. Additionally, the sensitivity, long-term stability and a wide linear range of humidity concentrations was achieved, as reported by Rathi et al. [55].

Miniaturized magnetoelastic humidity sensors. A humidity-sensitive thin film, made of nanodimensionally porous TiO_2_, was applied for the assembly of remote query humidity sensors, which is described by Varghese et al. [56]. The honeycomb structure, with a pore size of approximately 80 nm, makes the ceramic layers suitable to quantitatively trap moisture. The sensors were tested in a 50 cm diameter cylindrical humidity chamber (1 m length), about which a ten-turn pick coil was wound. Results demonstrated the resonant frequency in response to high and low relative humidity levels, of 60% and 2%, respectively. Experimental data showed the reversibility of the magneto-elastic tools, with the resonant frequency measured at increasing and decreasing humidity levels. A deviation from the response linearity, presumably was related to the rate-limited diffusion times into the ceramic materials. This effect represented a limitation of the sensors, successfully solved by using aluminum thick films (see Figure 1b and reference [57]). Using this latter, the impedance variation resulted higher than that recorded in literature [58] for other ceramic nanostructured sensitive layer, more than three orders of magnitude, ranging from ~20% to ~90%, with a response time of ~95 s. The miniaturized remote query magneto-elastic micro-sensor arrays could be also assembled as a multi-parameter device, dedicated to the simultaneous monitoring of pH, temperature and pressure values. In the case of pH detection, the responsive poly(acrylic acid-co-isooctylacrylate) film was synthesized at 70 °C by free radical copolymerization of acrylic acid and isooctylacrylate, on the 2826 MB ((Fe_40_Ni_38_Mo_4_B_18_) transducer surfaces [59]. The calibration curve, for a polymeric thickness layer of 0.7 μm measured at 23.1 °C, showed a response profile for pH, decreasing from 7.5 to 1.3 with a resonant frequency shift of approximately 0.6% pH. The sensor platform was wireless and the output signal was recorded through a remotely located pickup coil. A remote query ammonia wireless sensor, combined with a free-standing magneto-elastic thick-film, coated with poly-acrylic acid-co-isooctylacrylate, was also reported in literature [60]. In this case, the mass of the polymer layer influenced the resonant frequency of the ferromagnetic magneto-elastic support, remotely quantifying the NH_3_ concentration levels, without physical connections to the devices. The sensor linearly recorded the ammonia concentration level below 0.8 vol.% and logarithmically detected the highest ammonia concentration values [60]. 

For this kind of miniaturize sensor, there are no specific applications (in terms of air quality monitoring) carried out in famous museums, archives, libraries and galleries, as reported above for the other kind of humidity sensors. Magnetoelastic humidity sensors and ammonia probes are widely applied in food chemistry quality control for bacteria (i.e., *Staphylococcus aureus*) detection, rather than in the field monitoring campaign, dedicated to the conservation status of CH. 

**Figure 2:**
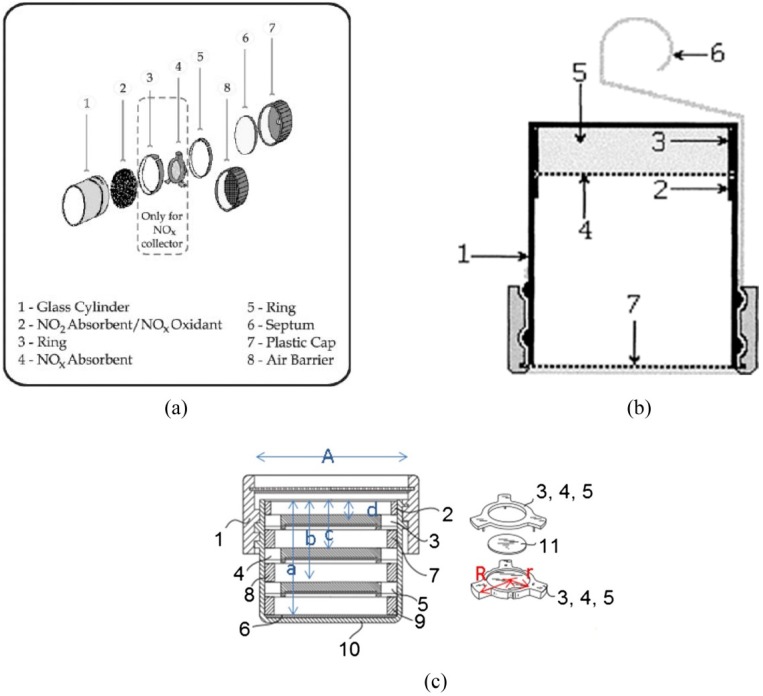
(**a**): Exploded scheme of Analyst NO_2_ and Analyst NO_x_ passive samplers [62]; (**b**): Structure of the ‘Analyst’ sampler, improved by Bertoni et al. [66] for BTX; (**c**): Exploded scheme of the multi-pollutant sampler, according to the literature [67].

Miniaturized diffusive samplers for gaseous pollutants. The measurement of gaseous and particulate pollutants in museum, libraries, galleries and archives is important to assure acceptable conditions related to the protection of CH surfaces. Diffusive miniaturized samplers (Figure 2a) were developed for the assessment of various gaseous pollutants [61], as a simple and less expensive alternative to macroscopic active sampling tools, reported in reference [61] and called denuder systems. If compared with the active samplers in which air is collected using a pump, diffusive samplers bring the pollutants into contact with the collector only by a spontaneous diffusion process. The first assembly of two badge-type passive samplers, having different lengths, was realized for the determination of NO_2_ and SO_2_ [62]. The samplers contained a filter membrane barrier to damp out eddy motions and promote pollutants diffusion from the environment to the selective pollutant trapping medium. The determination of NO_2_ was performed using a carbon filter coated with sodium carbonate (1% (*m/v*)) and glycerin (1% (*m/v*)) in a water/methanol (50/50) solution. The results, expressed as a ratio (R) between the 48 h and the consecutive 24 h based periods, demonstrated good stability for the collected pollutants (especially R = 0.98 ± 0.05 for nitrite and R = 1.08 ± 0.09 for sulphate). The detection limits were: 5 ppb for NO_2_ and 10 ppb of SO_2_ [63,64]. The accuracy (expressed as percent relative error) resulted better than about ±10%, when compared to denuder technique, combined with the coefficients of variation of 5.5% and 6.5%, respectively. De Santis et al. [65] improved the geometry of the passive sampler, introducing the Analyst tool (Figure 2b) previously modified by Bertoni et al. [66] for the determination of BTX (Benzene, Toluene and Xylene). The uptake rate of NO_2_ of 11.7 mL/min is in agreement (within 5%) with the reference values. The measured uptake rate for NOx/_g_ (where NOx/_g_ = NO + NO_2_) was quantified in the experiments, involving different levels of concentrations of NO_x_ and compared with the ChemiLuminescence (CL) measurements. The precision of the measurements resulted better than 5%. The accuracy of the data was ±20% of the value, this latter measured by CL. The collected field campaign results demonstrated that the NO_x_ and NO_2_ samplers meet the data quality goal, requested by the first EU Directive 1999/30/EU for these nitrogen based pollutants. Considering the data quality goal, De Santis et al. [65] decided to apply the Analyst® (Marbaglass, Rome, Italy) in the Uffizi Gallery (Florence, Italy) for the quantification of O_3_, SO_2_, NO_2_, NO, HNO_2_ and HNO_3_ gaseous pollutants. The monitoring campaign was carried out during eight periods, starting from March 2001 to February 2002. Results showed that the Indoor/Outdoor (I/O) concentration ratio represented a good indicator of both penetration from outdoors and reactivity for given species, toward the museums surfaces. The understanding of the space-time evolution of (I/O) pollutants is fundamental for understanding the effects of damage on works of art.

Experimental results showed that SO_2_, O_3_ and HNO_3_ were invariably lower indoors, as expected considering their chemical reactivity. NOx and NO_2_ resulted quite similar to the corresponding outdoor values. Instead, the indoor HNO_2_ concentration levels were largely in excess, compared to the values recorded, outside. Authors explained this effect considering the indoor heterogeneous production of nitrous acid, from NO_2_ and aqueous vapor (adsorbed on the surfaces). Since indoor environments are generally characterized by high surface-to-volume ratios (if compared with the outdoors), the heterogeneous formation of HNO_2_ is highly favored. The HNO_2_ and HNO_3_ concentration profiles resulted more difficult to interpret because their production (from NO_2_) and their chemical reactivity (e.g., deposition to walls) resulted particularly complex, when using data averaged over such a long period. Mean nitric acid (HNO_3_) concentration values varied from 0.7 to 12.2 mg/m^3^. Furthermore, the authors [65] found high I/O ratios for HNO_3_, meaning that HNO_3_ exhibited a consistent indoor source, while HNO_2_ showed a high reactivity, mainly due to the inner deposition on the museum walls and surfaces. Adsorbed HNO_2_ on art work objects generally induces photo-oxidation phenomena, provoking oxidative stress (due to the production of oxygenated radical species (ROS)) on art work support. Additionally, the acidity of CH surfaces significantly increases in the presence of the adsorbed nitrogen based acids (mainly nitric and nitrous acids). Acidic pH catalyzes several deterioration and degradation processes on CH surfaces, contributing to the aging of the art work materials. For this purpose, the monitoring of gaseous pollutants, in museums and Art galleries, provides useful information for the best strategies to select toward the conservation of CH. Recently, Vichi et al. [67] reported about a novel multi-pollutant diffusive sampler (Figure 2c) for HNO_2_, HNO_3_ and NO_2_, applied in different libraries, located in Switzerland in Bern, Geneva, and in Prague in the Czech Republic.

Experimental results showed lower I/O values for NO_2_, meaning that a greater penetration of pollutants indoors occurred in the naturally ventilated library, rather than in the filtrated archives. The indoor concentrations of HNO_3_ resulted very low probably for the highest deposition rate, on the indoor surfaces. HNO_2_ concentration values were usually lower than that recorded in outdoor environment, indicating that HNO_2_ was heterogeneously produced. The results showed high reproducibility of the new multi-pollutant sampler (according to EU directives), meaning that the newly developed tool can be used in archives and libraries, as a valid alternative to the Analyst®, allowing to completely map the concentration levels of indoor pollutants. A significant improvement of the analytical performances of Passive Air Sampler (PAS) has been also carried out by McLagan et al. [68], for the quantification of gaseous Hg. Precision, based on 378 replicated experiments and performed at multiple sites resulted in about 3.6 ± 3.0%, confirming excellent reproducibility. The adjustment of the calibration data for temperature and wind speed should be used, especially if conditions are highly variable or deviate considerably from the average of the deployments (9.89 °C, 3.41 ms^−1^), reported by McLagan et al. [68]. Overall, the study demonstrates that the sampler was capable of recording background gaseous Hg concentrations across a wide range, in indoor and outdoor environment.

Portable sensors for particulate matter collection. Most of the Particulate Matter (PM) sensors are based on the physical light scattering principles. General overview on the instrument performances, based on light scattering, can be found in several review articles [69]. Actually, there are several commercially available PM samplers, with prices, ranging from 10 to 1000 EUR. They exhibit excellent results obtained by exposing the sensor to PM concentrations, mainly come from to the tobacco smoke and the diesel exhaust. Its small sizes (25 mm × 21 mm × 2 mm) guarantee easy integration into a portable platform for the detection of fine particles (mainly PM_2.5_ and PM_1.0_). The sensors have an air-microfluidic circuit that separates the particles by size, then transports and deposits the selected particles using thermophoretic precipitation on the surface of a microfabricated mass-sensitive Film Bulk Acoustic Resonator (FBAR) [70]. The mass-loading of the FBAR causes a change in its resonant frequency and the rate of the frequency change corresponds to the concentration of the uptake particles. Experimental results demonstrate a high sensitivity of 2 g/m^3^, up to 10 min of integration time. White et al. [71] reported on a miniaturized Micro Electro-Mechanical System (MEMS) for PM that employed the deposition of particulates from a sample stream onto a 1.6 GHz FBAR, via thermophoresis. The quantitative determination of the mass deposited was carried out by measuring the resonant frequency shift of a Pierce oscillator. Real-time measurements, made in an environmental chamber over several weeks, showed excellent correlation with the responses of other commercial aerosol instruments. An added mass of 1 pg could be detected and the Limit of Detection (LOD) was 18 μg/m^3^. This latter device weighs 114 g, has a volume of approximately 245 cm^3^, consumes less than 100 mW and would cost less than $100 USD.

An interesting application for a type of PM portable device was carried out in the Museum of Capodimonte in Naples, one of the most important museums in Southern Italy [72]. In this monitoring campaign, the authors selected:

Room 8 (90 m^2^) because there are two important works of art—“La Trasfigurazione di Cristo” by Giovanni Bellini and the “Ritratto del Vescovo De’ Rossi” by Lorenzo Lotto;

Room 11, larger than room 8, where the works of art “Danae” by Tiziano and the “Soflon” by El Greco, are located;

Room 17 where the valuable “Parabola dei Ciechi,” by Bruegel il Vecchio, is preserved.

For these cases of study, the authors applied the Dust Scan Scout Aerosol Monitor, a continuous dust analyzer. The measurement method concerns the light scattering, where air flows with a rate of 2 L/min, through the sampling head analyzer having the filters for PM_10_ and PM_2.5_. After the sampling head, the flow enters in the detection chamber where particles travel orthogonally to a laser light source (670 nm) and to the corresponding receiving system, consisting in an optical sensor. The analytical performances of the PM light scattering analyzers are: the concentration levels of total PM (PM_total_ = PM_fine2.5_ + PM_10coarse_) ranging from 0 to 0.5 mg/m^3^, with an accuracy of ±10% and a precision of 0.002 mg/m^3^. The sampling time is of 30 sec. The experimental results demonstrated that the PM_10_ concentrations were particularly high, significantly above the law limit, established by *ISPRA (117/2010 ISBN 978-88-448-0451-0 for the air quality of the indoor environment)*. Minimum concentration levels for PM, were always recorded during:

- days of closure to public, indicating that the presence of visitors was the main contribution to the indoor particulate matter, especially when people opened the doors, or their presence determined the resuspension of dust, previously deposited on the floor:
- or during the winter campaign (carried out, from March to May 2007).

The PM_2.5_ concentration usually resulted above 20 μg/m^3^ (that represents the law limit, established by *ISPRA 117/2010 ISBN 978-88-448-0451-0, for indoor environment*); the Outdoor and Indoor average concentration ratios (I/O) were estimated of 0.59 for PM_10_ and 0.64 for PM_2.5_. The lowest I/O data demonstrated that the main contribution to the indoor solid matter, come from the anthropogenic outdoor sources and the PM_2.5_ fraction was the most relevant component to the PM_total_. According to these experimental results, the total PM concentration levels could be attenuated, introducing an air circulation system, containing specific particulate matter filters. Reducing the PM concentration levels, implies to minimize the deposition effects of pollutants, on the Art Work objects and surfaces. It is very well known that PM is a vector/carrier of adsorbed gaseous pollutants and therefore, when PM deposits on Art Work surfaces, also the adsorbed pollutants arrive on CH surfaces, starting their deterioration events based on chemical aggressive mechanism reactions. As a further example of PM monitoring in the indoor environment, a 24-h size-segregated PM cascade impactor (Sioutas PCIS, SKC Inc., Eighty Four, Township, PA, USA, reported in Reference [73]) was assembled in the refectory (located in Milan, Italy), where the “Last Supper” painting (one of Leonardo Da Vinci’s most famous artworks) is preserved. The monitoring campaign was organized for one year, starting on December 2009 until November 2010. During this time, air samples were collected at 1 m directly below the painting and a few centimeters from the wall surfaces (where the painting is hanging) [73]. Using these sensors, experimental results showed that PM_2.5_ dominated the indoor particle levels, with organic matter (mainly Organic Carbon, OC), as the most abundant specie. The mass balance for OC implies two different components: inner and outer organic matter. The experimental results demonstrated that gasoline vehicles, urban soil and wood-smoke (i.e., the outdoor sources) only contributed to an annual average of 11.2 ± 3.7% for the indoor OC component of PM_2.5_. This means that the outdoor sources contributed as trace levels, in the OC fraction. OC showed a relatively low outdoor infiltration ratio but an important indoor source, mainly fatty acids and squalene. This large reduction of the outdoor infiltration, was mainly attributed to the efficacy of the deployed ventilation system in removing particles. Minimizing the particles in museums and art galleries means that wall paintings and other art work objects can be preserved for a long time, reducing degradation events due to the delivery of pollutants by PM carriers.

**Figure 3:**
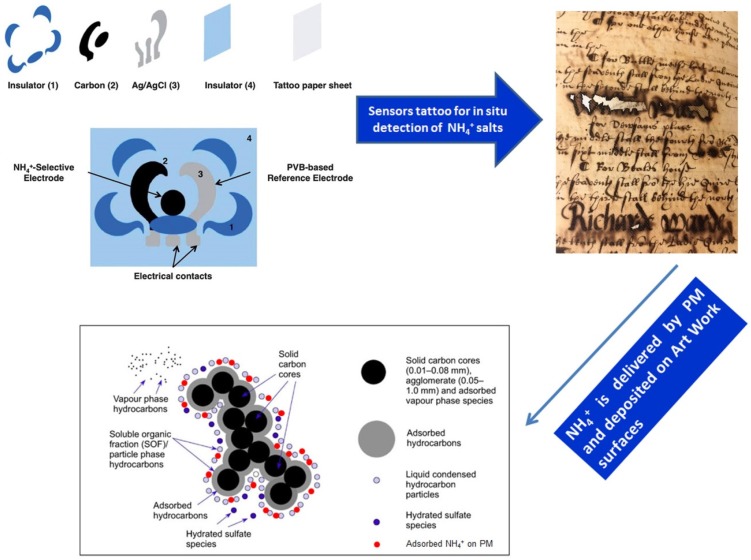
Layer-by-layer fabrication of the potentiometric tattoo sensor, as reported by Guinovart et al. [76]. The authors, display a cartoon to explain a future applications of tattoo sensors for the “*in situ*” detection of adsorbed pollutants and damages, on the CH surfaces.

Miniaturized Tattoo Sensors as new contact surface sensors. Recently, Bandodkar et al. [74] discovered and developed a new generation of non-invasive and in contact portable sensors, labelled as tattoo sensor devices (Figure 3). Bandodkar et al. [74] described the production, characterization and final application of temporary transfer tattoo-based sensors, equipped with a miniaturized wearable wireless transceiver (Figure 3), for real-time monitoring of several ions and molecules, useful to detect in health care field applications. There are several molecular probes interesting to quantify for their interaction with the CH surfaces. For example, pH and pI (this latter parameter, defined as pI but referring to ions activity, a_M+_) can be selectively measured, assembling the ISE (Ion Selective Electrodes) as described in Reference [75]. These electrodes are fabricated by coupling the screen printing and tattoo-transfer Nanotechnologies with solid-contact conventional/traditional ISE [75]. Printing technologies could be realized on different textile support, as: animal (wool), plant (cotton; cellulose paper; etc.), or synthetic (nylon, polyester) substrates. They possess inert chemical properties and yield stable operation for longer interval time. For example, an interesting application of these tattoo sensors on the CH field could be the monitoring of pH changes directly on deteriorated ancient paper manuscripts and damaged parchments. This in contact measurement could be extremely interesting to perform, especially before and after the restoration/conservation treatments, to evaluate the efficacy of the selected restoration treatments. Furthermore, the possibility to detect on CH surfaces, the amount of Na^+^, Ca^2+^, K^+^ provides a quantitative estimation of the inorganic salt concentrations, mainly responsible for the efflorescence/sub-florescence contamination damages. Among these environmental ions, the NH_3_/NH_4_^+^ ionic couple is very important for detecting CH and, for this purpose, Guinovart et al. [76] also developed ammonium based tattoo sensors. The quantitative analysis detected ammonium concentration in the range from 10^−4^ M to 10^−1^ M, with a near-Nernstian response, having a minimal hysteresis effect. Repeated mechanical deformations of the sensor showed negligible effects on the sensor tattoo output response signals.

This tattoo prototype could also be eligible for the monitoring of deposited ammonium salts (mainly as ammonium sulphate (NH_4_)_2_SO_4_, according to Ianniello et al. [77]) on art work surfaces, when delivered by fine particulate matter (mainly PM_2.5_). The ammonium tattoo sensor prototype seems to be eligible for in contact mode measurements of the ammonium salts, delivered by PM_2.5_ carrier, applying a non-invasive and non-destructive monitoring procedures.

**Scheme 1:**
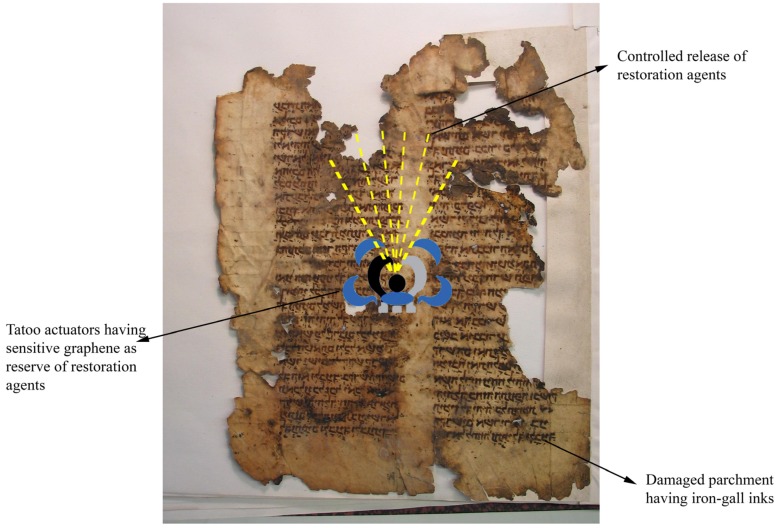
This cartoon (assembled by the authors of the review) represents the main future idea of the tattoo, as innovative actuator for in situ restoration of CHs, based on functionalized graphene nanomaterial, which act as reserve of restoration/consolidation chemical agents, suitable to release the selective Reagents in controlled way, on damaged Art Work surfaces. The damaged paper and parchment samples could provide the right clock for the modulated releasing of restoration agents, depending on the ΔpH, Δμ and other modification of chemical-physical parameters, induced by inner and/or outer deterioration events.

It is very useful to underline that the same tattoo sensor prototypes could work also as long-term reserves of restoration/consolidation chemical agents. The restoration/cleaning reagents could be properly released onto art work surfaces, only when significant changes in ΔpH, Δμ (where μ represents the ionic strength of the chemical environment of Works of Art) occur on the damaged historical surfaces (Scheme 1). In this case, the prototypes become future tattoo actuators, suitable for in situ restoration/remediation of damaged CH. The great future opportunity to combine smart tattoo sensors with highly performance tattoo actuators could provide both in situ diagnosis and *in loco controlled* restoration of damaged CH. The integration/combination of miniaturized tattoo devices (both sensors and actuators, in the same integrated chip) open a new scientific era/age of the movable Laboratory/Lab, dedicated to in situ diagnosis and *in loco* restoration of CH surface and objects.

On Table 2, a comparative summary concerning the analytical performances of the sensor prototypes presented in the review, is also reported. The monitoring of the conservation status of CH certainly represents the main point to induce restorers and conservator scientists to choose the best strategies for the restoration of art work objects (the need to preserve for a longer time). The best analytical performances (especially in terms of sensitivity) were observes in the presence of graphene derivatives, as sensitive layers of the resulting transducer tools. In the case of the sensors dedicated to the collection of the environmental pollutants, the activated carbon filters result as the most highly performing devices. The environmental samplers for gas and particulate matter pollutants could also be improved, especially in terms of sensitivity and lowest detection limits, using nanostructured carbon based filters.

Finally, on Table 3, a description of the most important field monitoring campaigns, carried out in the most famous museums and prestigious Art galleries, has been also reported.

To conclude the first section of the review, a new emerging sensor category has been described, concerning the imaging sensor devices, recently applied in CH field. The creation of 3D models for indoor heritage and outdoor archaeological areas provides a precise analytical method suitable to capture and digitally model the fine geometric details of such art work objects. Digital libraries, documentation and preservation are required, to preserve the worldwide CH, because on-going wars, natural disasters, climate global changes and human negligence (mainly environmental pollution) destroy the Art Work surfaces, areas and objects. In particular, the museums, libraries, archives, galleries and archeological areas have received a lot of benefits, applying the most recent imaging devices and technology. 3D data system acquisitions represent a critical component to permanently record the form of important art work objects so that, in digital form, they can be made usable by future generations. During the last decade, a large number of researchers, in the world, realized very good quality digital models. Among all of them, a recent 3D application model based on new TeraHertz (THz) imaging approaches has been developed. In particular, pulsed THz reflection imaging provides highly performances in terms of CH objects acquisition. In reflection method, portions of the THz pulse can return from any structural interface that exhibits a significant change in its refractive index. In addition, 3D-THz reflection images also provide useful information on the spectral or material chemical composition of CH samples, proportionally to modifications on the spectral content of the reflected THz pulses. Today, the most advanced commercial TD-THz instruments are portable/movable tools, equipped with interchangeable fiber-optic coupled remote scanning sensors. This new generation of apparatus provides the short-pulse laser, optical delays and signal processing to be deployed in a 19-inch rack-mounted control unit, while the THz transmitters and receivers can be remotely located and are freely positioned. These sensors can be used to scan large stationary art work objects/surfaces using either robotically controlled gantries, crawlers or hand-held devices.

According to these relevant performances, recently in the prestigious Uffizi Gallery in Florence (Italy), a pulsed THz scanner was applied during the restoration of the Politico di Badia [78], by Giotto di Bondone. THz miniaturized devices were mainly used to study the presence of the gold leaf under the pigments, of metal in some of the symbolic elements and also for the investigation of the chemical composition of the painting support. These panels were assembled and designed by carving both the painting surface and frame from a single piece of wood. The surface was then smoothed with a layer of gypsum, covered with canvas and more gypsum and then painted with organic pigments. All of these painting layers were not detected by using the current non-destructive technologies. The pulsed THz approach revealed these painting layers. The profiles of each painting layer were successfully detected, allowing the high resolution imaging of the canvas and the tool marks in the wood. For the first time, 3D images in the highest resolution, were acquired for the Uffizi paintings, only applying the THz scanner techniques. In this case study, the THz technique was performed with a non-contact, nondestructive imaging mode [78].

Recently, other researchers at the Louvre in Paris (France) are successfully examining, with THz scanner techniques, the wood grain of panels to perform precise dating and the evaluation of fragile written documents, avoiding the damages of the cellulosic supports. The availability of pulsed THz systems that are portable and exhibit fiber-optic coupled heads (useful to allow imaging of stationary objects) provides a great opportunity to perform in situ diagnosis, before and after the restoration/consolidation treatments, on damaged Works of Art.

## 3. Section II: Mobile Tools Integrated with ICT/IoT, for the Assembly of a Movable Laboratory

Conventionally, miniaturized and portable instruments are very well known and applied mainly for “*in situ”* diagnosis of the environmental damages, present on Art Work surfaces. Recently, Sciutto et al. [79] develop new systems apply lateral flow immunoassays to quantify two proteins (ovalbumin and collagen) in Cultural Heritage surfaces and objects. The new portable biosensors are equipped with chemiluminescent and colorimetric detectors. The chemiluminescent system displayed the best analytical performances, especially in terms of lower limits of detection, a wide linear range of concentration and a high sensitivity than that exhibited by the colorimetric detection system. To miniaturize the device, a disposable cartridge was realized ad hoc for in situ specific applications. The results highlight the enormous potential of these inexpensive, easy-to-use and minimally invasive diagnostic tools for conservators in the cultural heritage field.

The great opportunity to integrate portable sensors in the Smart Wireless Sensor Network (WSN) seems to be eligible for the Heritage scenarios protection. WSNs guarantee specificity, autonomy, self-configurability, lasting lifetime and mobility of the resulting portable apparatuses, suitable for in situ monitoring of CH. However, their services, protocols and architectures support new issues for protecting all the outdoor and indoor cultural heritage surfaces. According to these considerations, Rodríguez-Sánchez et al. [80] provide a new integrated modular system, consisting of three elements: a WSN, a “Central System,” and a novel platform named “Local Node Gateway.” It exhibits the coexistence of different wireless technologies in order to provide sensor functionalities, actuation for “*in situ*” restoration/consolidation, processing and communication performances, as described below, in details.

**Figure 4:**
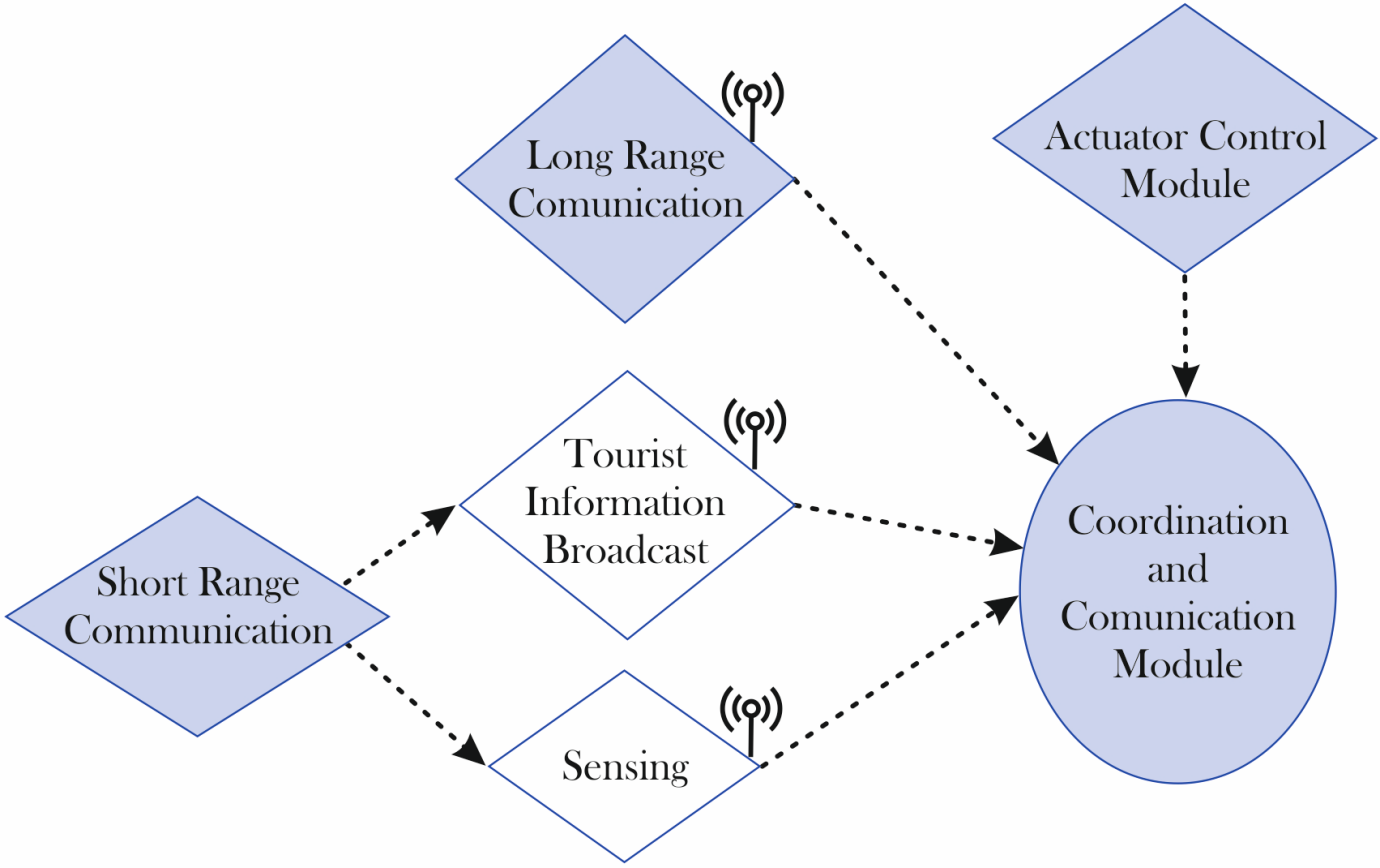
Block diagram for “Local Node Gateway”.

*(a)*  *Wireless Sensor Network (WSN).* The radio frequency communication is suitable for the monitoring of the Cultural Heritage environments, because is not limited by line-of-sight and the current technology allows implementation of low-power radio transceivers. ZigBee and Bluetooth are some standard protocols for short-range wireless communications. Especially, mobile phones and electronics devices adopt Bluetooth to send documents, files, images or documents for information of art work objects (especially before and after restoration/conservation treatments and consolidation procedures). The same authors [80] propose a WSN based on MICAz modules by Crossbow. They use MPR2400CA and MTS400 as transmission nodes, having different modules with several parameters, useful for the establishment of the conservation status of CHs such as: temperature, humidity, pressure, luminosity and two-axis acceleration (as reported in the first section of the review). This receptor node, based on MICAz and MIB510 boards; could allow to add new chemical sensors to detect additional parameters, as CO_2_ and other gaseous molecular pollutants and particulate solid matter pollutants.
*(b)*  *Central System (CS).* CS provides storage in a database, security and user interface to access to the environmental information, with capabilities of remote monitoring and storage in any place and any time. “Central System” consists of a web server, a database and a user interface. The server continuously receives data from the local environment to be processed and analyzed in real time. As the web interface, the same authors [80] have chosen Java. Java is a general-purpose, concurrent and multiplatform programming language. It accepts all the information from the environment using long-range wireless communication processes. These data are stored in the database using a web server and a database server, in order to perform environmental tasks. Considering that the environmental information is remotely monitored; some security mechanisms needs to be improved. A Firewall for protecting the resources of “Central System”, has been adopted by the authors according to the reference [80]. This control is over client users and “Local node Gateway” tasks. It was implemented using a user registering method, based on login and password to access into the system.
*(c)*  *Local Node Gateway (LNG).* All the required functionalities are in the “Local Node Gateway” with a single M2M GSM/GPRS device, as described in literature [81]. It has a processing and communication units, without external microprocessor and external embedded tools. It is designed in a modular, flexible, scalable and adaptable mode in order to provide sensing for analytical diagnosis, actuating for in situ remediation/conservation treatments, long range communication and broadcasting information to end-users. The advantages related to the power consumption, hardware platform size and cost, result three times better than other commercial systems. The proposed system it is already working at San Sebastian Church (Figure 4), Seville (Spain) since November 2008 [80]. The collected data are satisfactory to restorers and conservator scientists, who studied the experimental results to select the best strategies for the preservation of Cultural Heritage surfaces and objects. This working approach could be improved by combining the described technology with Internet services.

**Figure 5:**
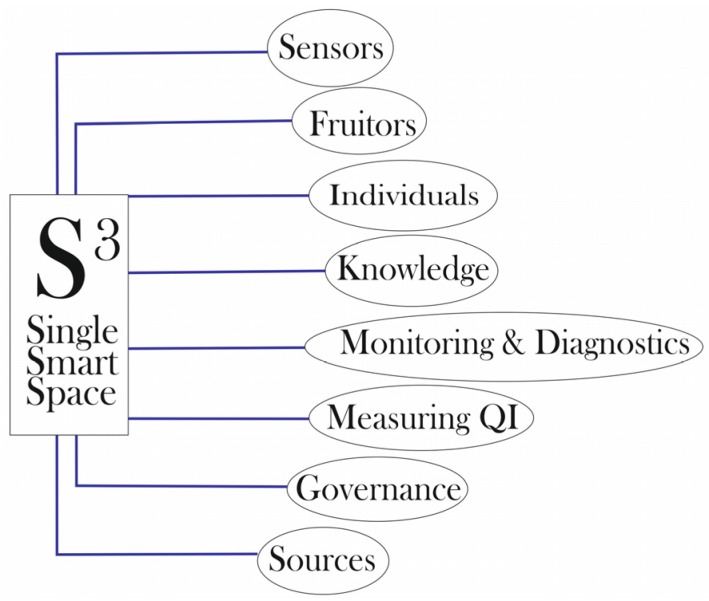
The single smart space S^3^ model.

For this purpose, the rapid development of Information and Communication Technology (ICT) and the growth of the Internet (Internet of Things, IoT) through high speed networks, network environments have even been changed from office oriented environments, based on business industries and public institutions, to the interconnection of digital electronics in home networks. Home network based applications are very diverse and the remote monitoring and control areas have been studied. Moreover, owing to the rapid growth of mobile technology, high performance smartphones are widespread and in increasing cases are utilized as terminal devices. Internet of Things (IoT) is a global infrastructure for the information society, enabling advanced services by interconnecting (physical and virtual) things based on existing and evolving inter-operable ICT [82]. Internet of Things (IoT) computing applied to the Art Work objects domain is an innovative discipline which consists of the application of smart sensor and actuator technologies on cultural, historical and archeological areas, strongly related to the development of systems able to be pervasive and ubiquitous with the definitive goal of re-thinking CH spaces [83]. IoT paradigm constitutes a powerful tool to enhance people fruition and enjoyment of such spaces (Figure 5). Thanks to ICT technologies, a cultural object can be effectively “dressed” of its context and juxtaposed into it, as reported in literature [84]. Intelligent and pervasive environments are characterized by a great number of devices and sensors/actuators that develop continuously and capture enormous amounts of data [85]. The Cultural Heritage domain represents a space where exchanged and produced data can be opportunely exploited by a set of applications and services in order to transform a static space into a “*smart*” environment [86]. By means of innovative technological applications and location-based services it is possible to shorten the distance between cultural spaces and their visitors, nowadays determined by the purely aesthetic and essentially passive fruition of cultural objects. Technology can become a mediator between visitors and fruition, an instrument of connection between people, objects and spaces to create new social, economic and cultural opportunities [87,88,89]. Today is no doubt that Internet of things is the next evolution of Internet: as Internet 2.0 has been represented by the world of social networking [90], relationships that happen in the global net and become shared information, Internet 3.0 is going to be the extension of Internet to the world of physical objects or things [91]. The challenge is to permit to the things to participate to a new extended social network and, then, to interact with other objects and with humans [92,93,94]. Also, we are going to enter in the era of ubiquitous computing and of Big Data collected by smart and advanced sensors/actuators [95,96,97]. Big numbers have a counterpart in the budgets of ICT companies and research institutes. The first role of ICT is to transmit intelligence to things, the objects of our daily life. Gartner, the world’s leading information technology research and advisory company, estimates that in next few years 26 billion objects will be connected [98], many of them in a global infrastructure. A report of ABI research considers this expectation cautious and expects about 30 billion of new physical connections. IoT refers to network connection of physical objects, using sensors, actuators and other control system tools and devices [99]. These components are able to register and transmit information about these objects. This amount of data can be analyzed to optimize products, services and operations. The new intelligent things, with decisional capabilities, will have tremendous energy saving potential for personal use (domestics and smart-home) and also, in a collective dimension (smart-city, smart-grid, protection of critical infrastructures) [100]. This massive number of sensors, actuators and other devices will be a powerful source of data; they have to be filtered, coordinated and elaborated. This new scenario will define a new Information and Communication Technology. The Big Data revolution exploded with the ever–increasing diffusion of social networks and will be enforced with the arrival of IoT: for the first time, companies will be able to discover new opportunities for studying, in a more effective manner, the behaviors of their customers [101]. So, companies will be able to evaluate the perceptions of their brands, services and products, especially when restorers and conservator scientists need to compare and to share results concerning the performances of new products and chemical agents, dedicated to the restoration and conservation of original art work objects. In this sense, IoT represents a new paradigm in the world of ICT that involves vendors, service providers, system integrators and academic research centers. The new molecules of this new eco-system that is going to born are sensors/actuators [102] and movable devices. There is a high request for new ones in terms of power, precision and characteristics of captured data. So, IoT will open new scenarios in terms of physical contexts for new applications. One of these contexts is represented by mobile Laboratories (i.e., mobile Labs). The reference architecture [103] consists of a physically constrained environment, a mobile room/laboratory that can be transported. Inside this laboratory thousands of heterogeneous sensors/actuators can be activated to analyze critical objects, where measurements are to be extremely precise, fast and repeated several times (for reproducibility test).

At the state of the art, there are some solutions about the fabrication of new portable Lab, completely dedicated to the diagnosis of damaged cultural heritage. The most important and highly performing architectures, are: Non-invasive mobile NMR (Nuclear Magnetic Resonance spectrometry) [104] and Laser-based systems [105] for the structural diagnostic of artwork; non-destructive tools for in situ micro analysis assays of cultural heritage materials and MOLAB, a Mobile Laboratory for in situ non-invasive studies in arts and archaeology [106]. MOLAB is equipped with an array of state-of-the-art portable and noninvasive instruments for both point and imaging analyses. The spectroscopic point techniques, integrated in MOLAB movable architecture, are: XRF (X-Ray Fluorescence), micro-Raman, mid-FTIR (Fourier Transform Infrared spectroscopy), near-FTIR, UV-vis reflectance, UV-vis fluorescence, TCSPT (Time correlation single photon counting for fluorescence lifetime measurements). Until now, there are not portable/mobile apparatuses, devoted to the in situ restoration and preservation of damaged art work surfaces but the current movable labs work only in diagnosis of the CH damages.

The new proof of concept, concerning a mobile Lab also equipped by miniaturized actuators could represent a future challenge, in CH field. Especially, the raise of nanomotors, nano machineries and nano propellers could be eligible for in situ remediation, converting their chemical energy into their mechanical cleaning effects, on art work surfaces. For this purpose, miniaturized actuators could be integrated in micro-chip systems, combined with the wireless smart transmission of the signals, for fast communication technologies. For multi-sensors and multi-actuators systems, with a high number of sensors and actuators, coordinated via an intelligent device, the Research has not yet defined methodological solutions to be applied for data analysis [107]. In this context, the main requirements are the precision and the synchrony between sensors and actuators, coordinated by an evaluator that receives multi-sources data. Other requirements are real time reconfiguration of all devices and real time coordination between data capture tools and data analysis tools [108]. Given the needs of new precision levels, new approaches have initiated in terms of learning theories and data analysis tools. Traditional techniques of artificial intelligence have been also stressed in this direction. These efforts are going to converge to a new interdisciplinary approach to the machine learning, where data flows come from multiple and heterogeneous sources [109]. The goal is to forecast the presence of hidden factors, starting from physical, biased, evidences. Keywords in this new approach are casual analysis based on ordered sets of data or trajectories. New mathematical tools for data manipulation are going to emerge. Experimental results are relative to many sector of human knowledge [110], from bio-informatics and analysis of DNA, to forensics, to anomaly detection in information security, to the cooperative evolution of systems in biology and other cooperative contexts. New advanced engines for timed and spatially referenced data will appear and will be used to recognize and evaluate, in real time, what is happening in critical environments, where strong qualitative results are expected.

## 4. Conclusions

This review focus on new portable and movable smart sensors, mainly based on miniaturized devices suitable to perform “*in situ*” investigation of the conservation status of CHs. The new portable devices offer several advantages, as: (1) the in situ diagnosis of un-tangible and immovable cultural heritage surfaces and objects; (2) the great opportunity to in situ restore and preserve Art Work surfaces without damages, considering the assembly of miniaturized actuators, that result to be non-invasive towards the materials of which the cultural heritage are composed; (3) the great opportunity to collect big data systems, all interdisciplinary information, thanks to the ICT and IoT new generation technologies, necessary to rapidly spread information among end users. The highest analytical performances of these devices, are exhibited especially when engineered nanomaterials were used for the assembly of integrated sensor and actuator systems. A powerful tool to design the multidisciplinary connection between new sensor array technologies and the high amount of useful information, flowing from these smart portable devices, is represented by IoT and ICT emerging technologies. Especially IoT systems cover the fundamental role of connector between the chemical-physical, biochemical world and the information world, in order to amplify the knowledge but also the enjoyment, in presence of Cultural Heritage and Art Work areas, objects and historical surfaces. Sensors, actuators, smart nanomaterials, ICT and IoT systems are all suitable tools for the assembly of a mobile/portable Laboratory, extremely useful for “*in situ*” diagnosis and also for “*in loco*” remediation/restoration of CHs and Archeological places. The experimental results, acquired during the restoration events of Art Work objects, can be shared in real time in order to inform end-users about the origin of CH damages, their restoration and the selected preservation strategies.

## Figures and Tables

**Table 1:** Summary of the main challenges, research needs, limitation and drawbacks of the CH sensors.

Sensor Description	Challenges	Limitations and Drawbacks	Research Needs
Conventional unmovable technologies, located in research laboratories	To assemble portable technologies for in situ monitoring especially for un-movable and un-tangible CHs	Portable miniaturized sensors present the lowest sensitivity and the highest detection of limit if compared with those exhibited by the conventional tools, located in academic research laboratories	To improve the sensitivity of the miniaturized movable tools, by developing smart and highly efficient nanomaterials, applied for the sensor transducers modification
New generation of tattoo sensors	To contact mode measuring, directly applied on CH’s surfaces and walls	Lowest sensitivity and un-compatible materials, applied for the tattoo tools fabrication, toward the chemical composition of the Art Work objects	To develop highly sensitive nanostructured materials, suitable for the transducers modification/assembly, extremely compatible with the Art Work support.

**Table 2:** Comparison in terms of the sensitivity for the most performing sensors.

Portable/Movable Sensors	Sensitive Nano Material Layers	Sensitivity Parameter	References
*Miniaturized optical sensors humidity*	Reduced Graphene Oxide	0.22 dB/% RH	[49]
abry-Perot resonator			
*Miniaturized capacitive sensors humidity*	Graphene Oxide (GO)	up to 37,800%	[54]
*Miniaturized pH-transducers*	Acrylic acid/isooctylacrylate and 2826 MB ((Fe_40_Ni_38_Mo_4_B_18_) surface	0.6%/pH	[59]
*Passive samplers (PAS)*	Carbon paper filter, coated by 3.0 mM Na_2_CO_3_/0.3 mM NaHCO_3_, aqueous solution	5 μg/m^3^ (NO_2_)	[62]
NO_2_			
SO_2_		10 μg/m^3^ (SO_2_)	
*Particulate Matter and portable sensors*	Filter-pack systems	2 g/m^3^	[70]
Micro-fabricated mass-sensitive Film Bulk Acoustic Resonator (FBAR)			

**Table 3:** Summary of the most important monitoring field campaigns, organized in several famous national and international museums, libraries and galleries, discussed in the review.

Sensor Category Description	Indoor Environment, Selected for the Monitoring Field Campaigns	References
Crossbow mica2 motes, equipped with an MTS400CA data acquisition board and a mib520board (as the base station)	Museum called Fortaleza São Tiago, located in Madeira Island, (Portugal)	[50]
MEMS based technology has been applied, where: the temperature transducer is based on a semiconductor diode element; the humidity probe is based on a capacitive element	The Cloisters, the medieval branch of the New York Metropolitan Museum of Art (USA)	[52]
Analyst^®^ (Marbaglass, Rome, Italy), patented by CNR gas sensing devices	Uffizi museum and gallery, Florence (Italy)	[65]
Novel multi-pollutant diffusive sampler	Different libraries/archives, located in Switzerland in Bern, Geneva, in Czech Republic and also in Prague	[67]
Dust Scan Scout Aerosol Monitor, that is a continuous dust analyzer	Museum of Capodimonte in Naples (Italy)	[72]
24-h size-segregated PM cascade impactor (Sioutas PCIS, SKC Inc., Eighty Four, PA, USA)	Refectory in the city center of Milan (Italy), where the “Last Supper” painting, one of Leonardo da Vinci’s most famous artworks, is preserved	[73]
